# Knee Arthrodesis Affects Gait Kinematics More in the Ankle Than in the Hip Joint

**DOI:** 10.3390/medicina58060696

**Published:** 2022-05-24

**Authors:** Nele Wagener, Sabrina Böhle, Julia Kirschberg, Sebastian Rohe, Markus Heinecke, Pietro Di Fazio, Georg Matziolis, Eric Röhner

**Affiliations:** 1 Department of Trauma Surgery, Orthopedics and Plastic Surgery, University Medical Center Goettingen, Robert-Koch-Str. 40, 37099 Göttingen, Germany; 2 Orthopaedic Professorship of the University Hospital Jena, Orthopaedic Department Waldkliniken Eisenberg, 07607 Eisenberg, Germany; s.boehle@waldkliniken-eisenberg.de (S.B.); j.kirschberg@waldkliniken-eisenberg.de (J.K.); s.rohe@waldkliniken-eisenberg.de (S.R.); m.heinecke@waldkliniken-eisenberg.de (M.H.); g.matziolis@waldkliniken-eisenberg.de (G.M.); e.roehner@hbk-zwickau.de (E.R.); 3 Department of Visceral Thoracic and Vascular Surgery, Philipps University Marburg, Baldingerstraße, 35043 Marburg, Germany; difazio@med.uni-marburg.de; 4 Klinik für Orthopädie, Heinrich-Braun-Klinikum Zwickau, Karl-Keil-Straße 35, 08060 Zwickau, Germany

**Keywords:** bone defects, knee arthrodesis, gait analysis, gait kinetics, joint moments, pedography

## Abstract

*Background and Objectives:* No gold standard exists for treating persistent periprosthetic knee infections. Knee arthrodesis represents one treatment concept for extensive bone defects and extensor system insufficiencies. It has already been shown that knee arthrodesis leads to a significant reduction in one’s quality of life. The aim of this survey was to assess the influence of knee arthrodesis on the neighboring joints on the basis of gait analysis data. Our hypothesis is that the hip and ankle joints are negatively influenced by knee arthrodesis in the process of walking. *Materials and methods:* We performed six pedobarographic and four gait analytical measurements in six patients 2.4 ± 1.6 years after receiving knee arthrodesis at the operating ages of 69.1 ± 9.2 years. Gait analysis consisted of time–distance parameters/minute (number of steps, double support, cycle time, standing phase, step length, gait speed). A healthy group of test subjects (n = 52) was included as the control cohort. Gait analysis was conducted using a three-dimensional movement system and three force-measuring platforms to determine the ground reaction force. Foot pressure was measured using a pedography platform. *Results:* Five of six patients presented an incomplete rolling movement over the toes on the side that was operated on, presenting with a gait line ending in the forefoot area. All of the patients bore less weight on the side that was operated on. Three of six patients demonstrated a pathological gait line with a healthy opposite side ending in the forefoot area. All of the patients exhibited a reduction in gait speed and step length and a lower number of steps. All of the patients had a prolonged double support/cycle time. *Conclusions:* Isolated knee arthrodesis is associated with reduced forefoot repulsion, restricted movement on the side receiving the operation, and reduced movement in the ankle/knee joint. The hip showed norm deviations in the hip moment/angle. Knee arthrodesis causes reduced gait kinetics/kinematics. Our survey shows that the relative joint moments of the ankle joint and hip are often reduced. The ankle joint is more affected compared to the hip.

## 1. Introduction

Knee arthrodeses are the ultima ratio in cases where there are substantial bone defects following the removal of coupled knee endoprostheses [1,2], painful and unstable knee joint ligaments after periprosthetic infection with antibiotic-resistant pathogens [1,2,3,4,5], irreparable and destroyed knee joints, and multiple failed revisions [1,6]. Other indications include extremity-preserving tumor surgery, which is associated with an extensor system insufficiency, as well as neurogenic arthropathy with a lack of muscular stabilization in the knee joint [1,2,3,4,5]. These diverse indications explain the inhomogeneous patient population. The number of knee arthrodesis cases is in the single digits each year, even in larger hospitals. Currently, the indication for primary knee arthrodesis is clearly limited by the successful further development of knee arthroplasty in the last few decades. Nevertheless, 2% of all primary knee arthroplasties and 8% of all revision arthroplasties result in a knee arthrodesis, which explains the limited number of cases [4]. Knee arthrodesis is contraindicated in the presence of degenerative changes in the spine and in the adjacent joints [2,3,7]. The compensatory mechanisms to maintain mobility after knee arthrodesis include altered muscular balance, pelvic obliquity, ipsilateral hip abduction, and the dorsal extension of the of the ankle joint [1,2,3,4]. The surgical results of this knee fusion procedure differ greatly depending on the indication/surgical technique [1,5,8]. For example, modular implants are used for larger bone defects in order to minimize leg shortening [1,2]. The clinical results after knee arthrodesis in terms of mobility and physical activity are inferior to those of knee arthroplasty, but superior in terms of pain reduction [6]. Patients with knee arthrodesis show kinematic/kinetic deficits despite knee joint damage being surgically repaired [9,10]. Unfortunately, there is little biomechanical data on the resulting changes in the adjacent limbs [10,11,12,13]. This retrospective gait analysis study was intended to clarify the functional consequences of a knee arthrodesis on the lower extremities after an average follow-up time of two years. Furthermore, significant patient morbidity and poor quality of life could be exacerbated by bilateral pathological joint mechanics. Gait analysis data are necessary to make pathomechanisms transparent so that individual techniques/therapies can be improved to optimize the patient’s biomechanics in future knee arthrodesis operations.

## 2. Materials and Methods

### 2.1. Gait Analysis Patients

A total of six knee arthrodesis patients (n = 6): three women (n = 3) and three men (n = 3); operating age: 69.1 ± 9.2 years; the body mass index was 35.5 ± 9.1 kg/m^2^, corresponding to grade I and II obesity, were included in the study. The follow-up examination took place after 2.4 ± 1.6 years. Two out of six patients were dependent on a bilateral walking aid at the time of the gait analysis. Comorbidities included a toe amputation of digitus 2, a bilateral hip replacement, an ankle joint arthrodesis on the side of the knee arthrodesis, and a patient with post-polio myelitis status. The results of the knee arthrodesis patients were compared to those of a healthy group of subjects from the demographics data set “normal persons” from Novel (n = 52; left: 148 and right: 157 measurements), who weighed 65.2 ± 9.2 kg; however, we did not have access to age or gender information.

### 2.2. Data Collection/Analysis

We used Vicon 460, a three-dimensional motion gait analysis system with six infrared cameras (frame rate 100 Hz) and three force-measuring platforms (1× Kistler Instrumente AG, Winterthur, Switzerland, CH; 2× AMTI, Watertown, MA, USA, measuring frequency 1000 Hz) to determine the ground reaction force. A Plug in Gait (PIG) was used as a model to determine the gait parameters. We used the emed^®^c50 pedography system from Novel (Munich, D) for the foot pressure measurements. The sensor area was 395 × 240 mm. There were 3792 sensors, and there were 4 sensors per cm^2^ (measuring frequency: 50 Hz). The parameters were determined using the “emed professional” report for the entire foot and using masks for 10 zones on the foot (heel; metatarsus; metatarsal bones 1, 2, 3, 4 and 5; big toe, second toe, and toes 3, 4 and 5). The maximum force (% KG), peak pressure (KPa), contact time (% ROP) and contact area (cm^2^) served as parameters for the rolling process. To evaluate the gait analysis parameters, the relevant maximum/minimum values of the curves or relevant parameters such as angle, moment, and power were considered. The time–distance parameters included cadence, double support, cycle time, standing phase, step length, and gait speed. The ground reaction forces that were transferred to the floor when walking were recorded with force plates. The relative joint moments related to body weight and the joint angles of the pelvis, hip, knee, and ankle joint, were documented in the sagittal, frontal and transverse planes and were calculated using dedicated Nexus software for the gait analysis system based on a validated musculoskeletal model [14].

### 2.3. Statisticical Analysis

Currently, indications for knee arthrodesis are significantly limited, resulting in a decrease in the number of cases due to the successful further developments in knee arthroplasty. The individual patients with different comorbidities, as well as a gold standard subject who did not experience comorbidities, were compared with healthy subjects (NP) (n = 52).

## 3. Results

### 3.1. Pedography

**Figure 1:**
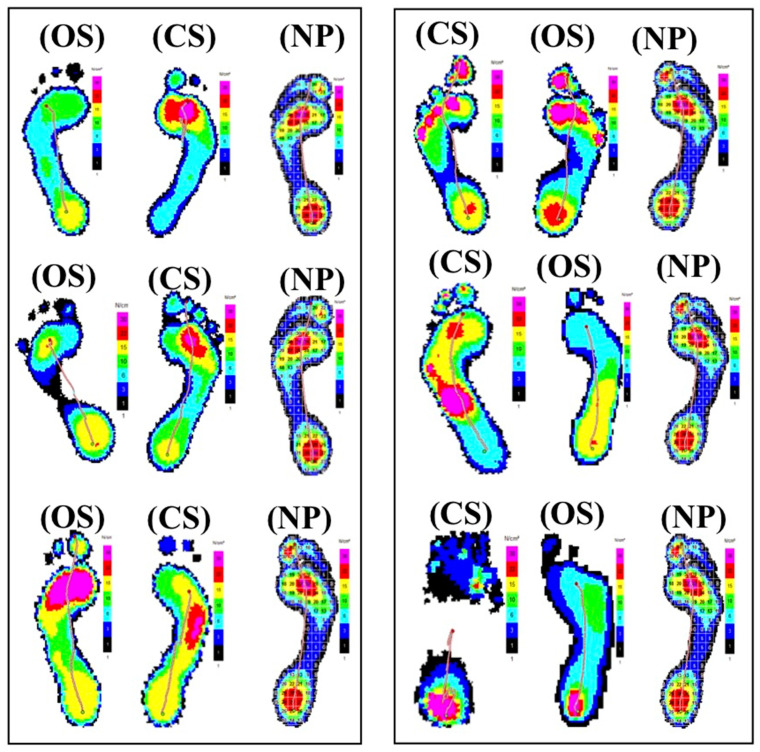
Comparison of maximal pressure images with dynamic foot pressure measurements; (OS): operated side; (CS): contrary side; (NP): normal person.

**Figure 2:**
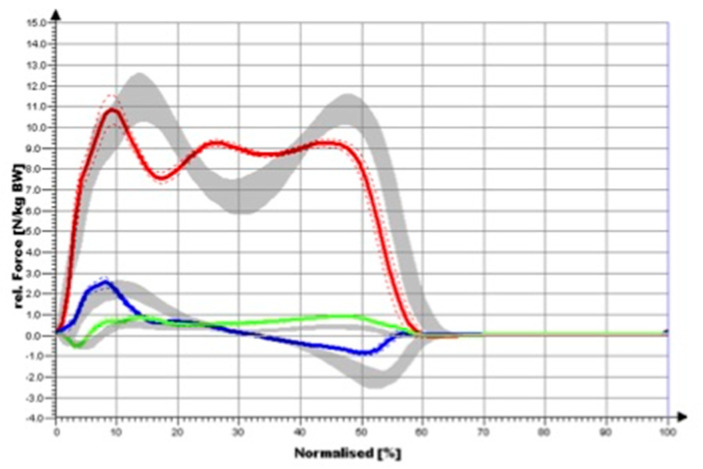
Three–dimensional vector representation of ground reaction forces during walking (OS): red; (CS): blue; (NP): grey.

The foot pressure measurements that were taken for the operated side (OS) were compared to a control foot of an (NP) with regard to rolling movement, gait line, and weight-bearing ability (Figure 1 and Figure 2). The foot pressure measurements of the control foot showed a gait line from the rear of the foot to over the toes, indicating that a complete rolling movement was possible. The maximum pressure pattern of the (NP) foot showed a maximum pressure distribution in the areas of the heel, metatarsal bones II–III, and the big toe. Five out of six knee arthrodesis patients showed no complete rolling movement over the toes on the side that was operated on and had a gait line that ended in the forefoot area. A total of 100% of the patients showed less weight-bearing ability on the (OS). Three of six patients showed a contrary side (CS) with a pathological gait line that ended in the forefoot area. Three of six patients showed that they had a maximum pressure pattern in the forefoot area on the CS, whereby the main load in one patient extended over the entire forefoot area including the big toe. Two out of six patients had the maximum pressure pattern in the metatarsal area on the contrary side. One patient displayed the maximum pressure pattern on the CS over the heel.

### 3.2. Gait Analysis

The gait analysis highlighted a reduction in the cadence, the gait speed, and the step length of the OS/CS in 100% of the patients compared to healthy normal people (NP) (Table 1). A total of 100% of the examined patients showed OS/CS with a prolonged double support and cycle time. Three out of four patients demonstrated a reduced standing phase on the OS, whereas it was minimally prolonged in one patient. A total of 100% of the patients showed an extended double support, standing phase of the CS, and cycle time.

**Figure 3:**
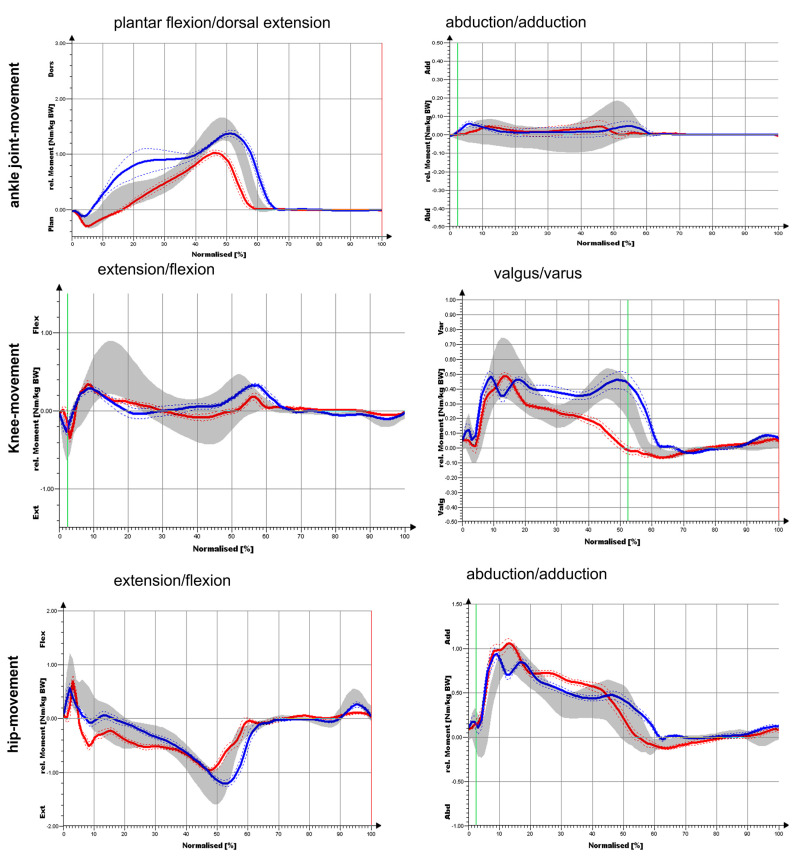
Joint moment progression of the ankle joint, knee and hip during a complete gait cycle in the sagittal plane (flexion/extension) and frontal plane (abduction/adduction).

**Figure 4:**
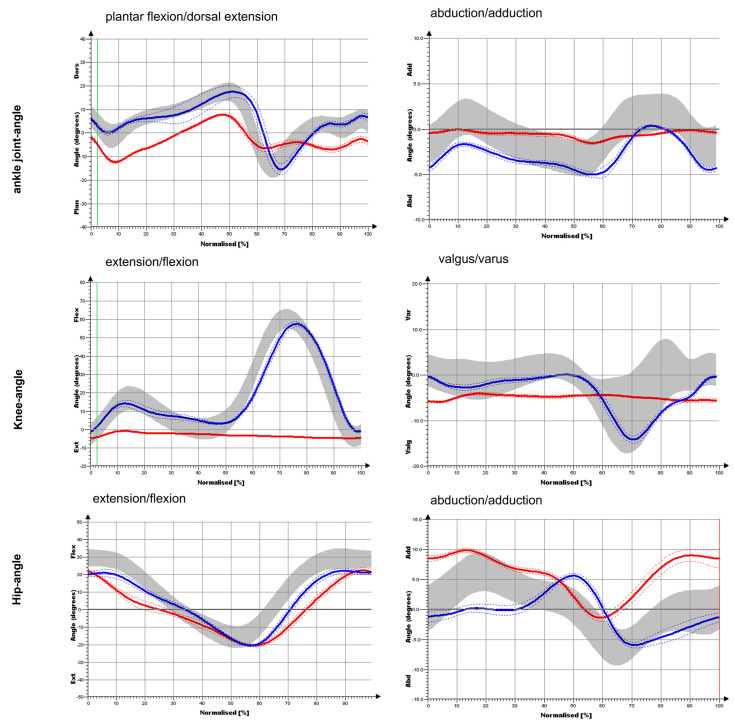
Joint angle progression of the ankle joint, knee and hip during a complete gait cycle in the sagittal plane (flexion/extension) and frontal plane (abduction/adduction).

Our gold standard case who did not have any musculoskeletal comorbidities only had a knee arthrodesis and was compared to the healthy normal subjects. The gold standard patient was able to step up with the heel relatively forcefully (1st peak), but did not push off forcefully with the forefoot (2nd peak) (Figure 3). Compared to NP, clear restrictions were detected not only for the knee joint but also for the hip joint and the ankle joint during the course of movement. While plantar flexion in the sagittal plane was particularly limited compared to (NP), there was almost no movement in the leg that was operated on in the frontal plane (Figure 4). The ankle joint on the CS was comparable to the values of the NP. For the knee angle, there was virtually no movement on the side that was operated on in either plane. On the opposite side, the values obtained for the knee joint were comparable to the NP values. Regarding the frontal hip angles, there was a shift towards an increased adduction posture for the OS and a shift towards abduction for the CS in the touchdown area. The sagittal hip angles were comparable to the values of the NP on both sides. In the sagittal plane, the ankle-joint moment was largely identical to that of NP (Figure 4). In the frontal plane, the ankle-joint moment was greatly reduced on both sides. In the sagittal plane, the knee moment was greatly reduced on both sides. In the frontal plane, the knee moment in the heel strike area was greatly reduced for both sides (reduced valgus moment). For the CS, the further course of the curve was largely comparable to that of NP, while the course on the operated side was significantly altered. In the sagittal plane, no considerable differences were observed in terms of hip moment compared to NP for the OS and CS. It was only the extension moment that was somewhat limited. In the frontal plane, no substantial differences were found in NP for the OS and the CS. Thus, hip moments demonstrated the smallest deviations compared to the knee joint and ankle joint.

## 4. Discussion

With this survey, we were able to demonstrate that knee arthrodesis is associated with a reduction in the kinematics of the OS and the CS. The dynamic foot pressure distribution revealed a substantially reduced load on the side that was operated on in 100% of the patients, a finding that can be attributed to plantar flexion weakness in the ankle joint. This plantar flexion weakness was also evident in the gait analysis results of our gold standard, which revealed no other musculoskeletal pathologies and who was followed up after 4.7 years, confirming that knee arthrodesis reduces gait kinematics more in the ankle joint than in the hip joint. Similarly, reduced abduction/adduction was found in the frontal plane on the ipsilateral side. Knee arthrodesis leads to the ipsilateral ankle having an altered joint geometry accompanied by a reduction in strength. This results in the requirement of increased effort when walking. These limitations result in a severely restricted quality of life for knee arthrodesis patients. On the OS, the frontal hip angles showed a shift towards an increased adduction posture and a shift towards abduction on the CS. It can be assumed that as the knee arthrodesis ages, there is an even greater reduction in the ankle joint and hip angle. The gait analysis carried out by Marshall and colleagues showed no deviation in the cadence, stride length, and average velocity variables in two knee arthrodesis patients compared to normal subjects, but a five percent reduction in the stance time on the side experiencing knee arthrodesis [7]. Our gait analysis measurements showed a reduction in the cadence, gait speed and step length on the CS/OS side in four knee arthrodesis patients compared to normal subjects (n = 52). In contrast to Marshall and colleagues, who showed increased plantar flexion in the ankle on the side experiencing knee arthrodesis, our measurement data revealed limited plantar flexion on the side that was operated on and mobility that was comparable to normal subjects on the opposite side. The subjects that were included in the work carried out by Marshall and colleagues showed excessive joint moments and a high range of motion in the ankle and hip joints. Our gait analysis measurements revealed minor deviations in the hip moments in contrast to the knee and ankle. Malley et al. confirmed our results by stating that their gait analysis of knee arthrodesis patients showed a significant reduction in gait speed and step length [13]. Hutchison et al. was also able to show that knee arthrodesis is associated with reduced plantar flexion on the OS and increased hip abduction on the CS [8]. Thus, the results of Hutchison et al. support our results. Furthermore, Giannini et al. demonstrated that knee arthrodesis caused increased hip migration and transverse pelvis rotation on the affected side and an increase in the extension of the opposite hip in gait analysis studies [10]. Our frontal hip angle measurements revealed a shift toward an increased adduction posture on the side experiencing knee arthrodesis and a shift towards abduction on the CS. However, subsequent follow-up examinations after three years would be necessary in order to document possible joint angle reduction progression in the ankle joint and in the hip. In our study, 100% of the patients showed that the healthy CS could not compensate for the pathological altered time/distance parameters. Due to the pathology of the gait cycle, it can be assumed that the musculoskeletal system of the side that was not operated on, will become diseased. However, Conway et al. stated that above-knee amputation is associated with an inferior functional outcome than knee arthrodesis [1]. If a progressive deterioration in joint angular function and persistent pain is observed, then above-knee amputation should be considered as an alternative to knee arthrodesis to avoid musculoskeletal system diseases on the healthy CS. The limiting factor of our study is that knee arthrodesis as an exceptional indication for non-recoverable bone defects/infections and represents a small inhomogeneous patient population with a correspondingly limited number of cases.

## 5. Conclusions

Our knee arthrodesis subjects with different comorbidities show significant limitations in terms of their cadence, gait speed and step length that are analogous to isolated knee arthrodesis without comorbidities. Consistently, all of the subjects showed reductions in not only their gait speed but also in the bilateral angle/knee joint and hip joint moment, with the ankle joint showing the greatest amount of impairment. The present study shows that indications for knee arthrodesis do not exclude the degeneration of the adjacent joints and should therefore be made based on a patient-specific basis by taking age and comorbidities into account.

## Figures and Tables

**Table 1:** Time–distance parameters; gold standard represents a sole knee arthrodesis.

	Cadence	Double Support	Cycle Time (OS)	Cycle Time (CS)	Standing Phase (OS)	Standing Phase (CS)	Step Length (OS)	Step Length (CS)	Gait Speed (OS)	Gait Speed (CS)
Normal persons	115.9	0.26	1.04	1.04	62.30	62.30	0.72	0.72	1.39	1.39
patient 1	78.85	0.41	1.55	1.50	62.60	63.92	0.45	0.38	0.53	0.53
patient 2	99.76	0.30	1.20	1.21	58.39	66.32	0.64	0.55	1.00	0.98
(gold standard)										
patient 3	80.19	0.40	1.50	1.49	60.69	66.43	0.43	0.50	0.60	0.61
patient 4	106.93	0.30	1.12	1.13	60.81	66.15	0.46	0.56	0.93	0.93

## Data Availability

Not applicable.
